# Gal-3BP in Viral Infections: An Emerging Role in Severe Acute Respiratory Syndrome Coronavirus 2

**DOI:** 10.3390/ijms23137314

**Published:** 2022-06-30

**Authors:** Valentina Gallo, Alyexandra Arienzo, Stefano Iacobelli, Valentina Iacobelli, Giovanni Antonini

**Affiliations:** 1 Department of Sciences, Roma Tre University, Viale G. Marconi, 00146 Rome, Italy; valentina.gallo3@uniroma3.it; 2 Biostructures and Biosystems National Institute (INBB), Viale delle Medaglie d’Oro, 00136 Rome, Italy; alyexandraarienzo@gmail.com; 3 MediaPharma Srl, Via Colle dell’Ara, 66013 Chieti, Italy; s.iacobelli@madiapharma.it (S.I.); vale.iacobelli@gmail.com (V.I.)

**Keywords:** Gal-3BP, virus infection, SARS-CoV-2, biomarkers

## Abstract

Galectin-3 binding protein (Gal-3BP) is a multifunctional glycoprotein involved in cell–cell and cell–matrix interactions known to be upregulated in cancer and various viral infections, including HIV-1, HCV, and SARS-CoV-2, with a key role in regulating the antiviral immune response. Studies have identified a direct correlation between circulating levels of Gal-3BP and the severity of disease and/or disease progression for some viral infections, including SARS-CoV-2, suggesting a role of Gal-3BP in these processes. Due to Gal-3BP’s complex biology, the molecular mechanisms underlying its role in viral diseases have been only partially clarified. Gal-3BP induces the expression of interferons (IFNs) and proinflammatory cytokines, including interleukin-6 (IL-6), mainly interacting with galectin-3, targeting the TNF receptor-associated factors (TRAF-6 and TRAF-3) complex, thus having a putative role in the modulation of TGF-β signaling. In addition, an antiviral activity of Gal-3BP has been ascribed to a direct interaction of the protein with virus components. In this review, we explored the role of Gal-3BP in viral infections and the relationship between Gal-3BP upregulation and disease severity and progression, mainly focusing on SARS-CoV-2. Augmented knowledge of Gal-3BP’s role in virus infections can be useful to evaluate its possible use as a prognostic biomarker and as a putative target to block or attenuate severe disease.

## 1. Introduction

**Figure 1:**
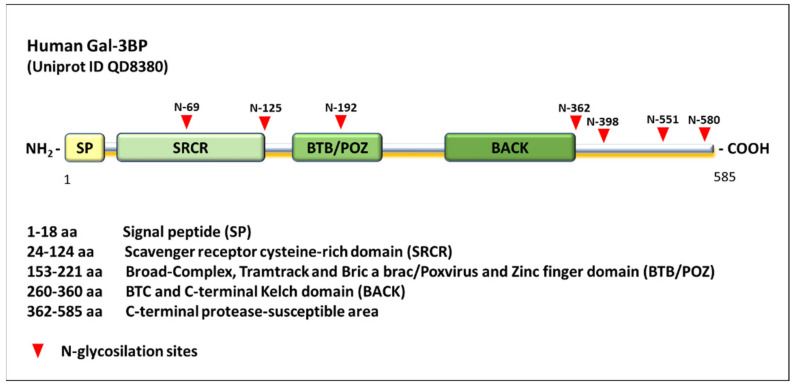
Human Gal-3BP’s structure consists of three main functional domains: an N-terminal scavenger receptor cysteine-rich (SRCR) domain, a BTB/POZ (broad-complex, tramtrack and bric-a-brac/poxvirus and zinc finger) domain and a BACK domain (BTB and C-terminal Kelch domain). In addition, there is an N-terminal 18 amino acid signal peptide that is cleaved after translation and a C-terminal protease-susceptible area.

Galectin-3-binding protein (Gal-3BP, Uniprot ID–Q08380), also known as tumor-associated antigen 90K or Mac-2-binding protein, is a multifunctional secreted glycoprotein encoded by *LGAL3SBP* gene [1,2] involved in cell–cell and cell–matrix interactions [3,4]. Gal-3BP is expressed in various cell types and found in serum and other biological fluids [5,6,7,8,9,10]. The full-length protein is constituted by 585 amino acids, but the molecular weight is 90 to 100 kDa due to extensive glycosylation [11,12]. After translation, the protein undergoes a proteolytic cleavage that causes the release of 18 amino acids from its N-terminal domain, leading the protein through the secretory pathway [1,13]. The structure of Gal-3BP consists of three main functional domains, including an N-terminal scavenger receptor cysteine-rich (SRCR) domain, a BTB/POZ (broad-complex, tramtrack and bric-a-brac/poxvirus and zinc finger) domain and a BACK (BTB and C-terminal Kelch) domain [14] (Figure 1).

The SRCR domain is known to be involved in protein–ligand interactions and to play a role in innate immune response [15,16]. The BTB/POZ domain has been demonstrated to have a role in transcriptional repression, cytoskeleton regulation, in targeting proteins for ubiquitination and degradation [17], and dimerization and oligomerization [18]. Indeed, Gal-3BP has been found not only as a monomeric protein but also in dimeric and oligomeric complexes, the latter being characterized by a ring-shape structure. The presence of these domains is consistent with the diversified functions exerted by the protein, together with the glycosylation state, and both are determinant for Gal-3BP oligomerization. Indeed, structural and functional studies demonstrated that the ring-shape conformation of the oligomer depends on the presence of all the three domains [12,19]. In addition, Gal-3BP contains a C-terminal cleavage site, presumably with regulatory functions, that might justify the existence of smaller forms of the protein (70 and 25 kDa) [1]. These features suggest that Gal-3BP can be finely regulated at diverse levels and that its regulation is based on the formation of multimers; the modification of protein conformation and length that mediate protein interactions; and affinity to different interaction partners, its activity, and turnover [20,21]. This highlights the complex biology of the protein, whose mechanisms of action could vary greatly depending on interactions with different molecular partners, on cell type, and disease considered. 

A multitude of epidemiological and clinical studies have documented that patients affected by pathological conditions such as cancer, viral infections, and autoimmune disorders frequently display supranormal levels of Gal-3BP [22,23,24,25,26,27], which are associated with unfavorable clinical outcomes [5,28,29,30,31,32,33,34]. On the contrary, low levels of Gal-3BP have been found to be associated with an augmented response to antiviral therapies [35]. Increased levels of Gal-3BP have been detected in diverse virus infections, including human immunodeficiency virus 1 (HIV-1), hepatitis B virus (HBV), hepatitis C virus (HCV), hantavirus, and dengue virus, and have been associated with an important antiviral significance [26,36,37]. Data have been presented to show that the activities of Gal-3BP in viral infections are mediated by the interaction of the protein with diverse intracellular and extracellular partners, which results in the induction of IFN and proinflammatory cytokines. In particular, interaction with galectin-3 [3], a multifunctional β-galactoside-binding animal lectin that is a modulator of both acute and chronic inflammation, is known to promote cell-to-cell adhesion and induce IL-6 and proinflammatory-signaling cascades [38]. Recently, increased plasma levels of Gal-3BP have been found in SARS-CoV-2-infected patients (COVID-19 patients) and, like in other viral infections, a correlation has been observed between increased levels of Gal-3BP, disease severity, and poor clinical outcome [39,40]. Furthermore, Kuśnierz-Cabala et al. have presented preliminary data suggesting that Gal-3BP could be involved in mediating outcomes of COVID-19 patients, such as lung fibrosis [39]. Some of the detrimental effects of increasing levels of Gal-3BP in COVID-19 patients could be due to its interaction with galectin-3 [41]. Indeed, the Gal-3BP/galectin-3 axis has been demonstrated to activate the antiviral innate immune responses by targeting TNF receptor-associated factors (TRAF6 and TRAF3) complexes, thus inducing expression of IFN and proinflammatory cytokines, including interleukin-6 [42], a known inducer of TGF-β signaling [43,44].

In this review, we investigated the most recent knowledge of the intricate biology of Gal-3BP in viral infections, with the aim of elucidating its mechanisms of action and the relationship with disease severity and progression, particularly in SARS-CoV-2 infection. The evaluation of Gal-3BP early in the course of the infection might represent a novel tool for prognosis and the discovery of new medicines for COVID-19 patients. 

## 2. Gal-3BP and Viral Infections

**Figure 2:**
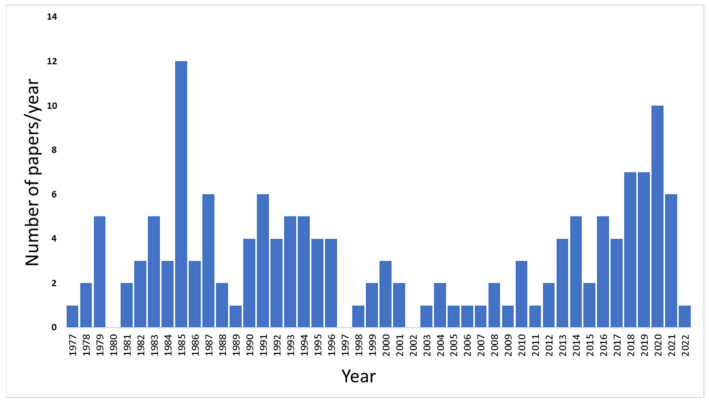
Number of publications per year (from 1977 to 2022) on Gal-3BP and viral infections. The search was performed using PubMed, Scopus, and Google Scholar databases, using the following key words: “(90K OR LGALS3BP) AND virus”.

The involvement of Gal-3BP in viral infections, its role in innate immune response, and the antiviral significance have been extensively investigated and documented, with a renewed surge of interest in the last 5 years (Figure 2).

The protein was originally found to be elevated in patients infected with HIV-1 [22,45]. Subsequent studies reported elevated levels of Gal-3BP in various viral infections, including HBV, HCV [36], hantavirus [37], and dengue virus [26]. These studies showed that Gal-3BP displayed an antiviral activity [46,47,48], which was related to the induction of IFN and proinflammatory cytokines, thus limiting virus expression and replication. Other studies showed that infection by influenza A virus (IAV), vesicular stomatitis virus (VSV), and herpes simplex virus (HSV) was associated with upregulated expression of Gal-3BP mRNA in mouse embryonic fibroblasts (MEFs) and in IAV-infected murine lungs [42]. Recently, increased levels of Gal-3BP have been found during SARS-CoV-2 infection and a correlation between Gal-3BP levels and COVID-19 disease severity has been observed [39,40].

The upregulation of Gal-3BP during viral infection has been demonstrated to be mediated by different cytokines that are commonly induced by virus infection, such as interferons (i.e., IFN-α, IFN-β, and IFN-γ) [47,49,50,51,52], tumor necrosis factor alpha (TNF-α) [53,54], and by double-stranded polynucleotides [55]. However, the mechanisms of action underlying its activities in virus infections are still poorly understood.

**Figure 3:**
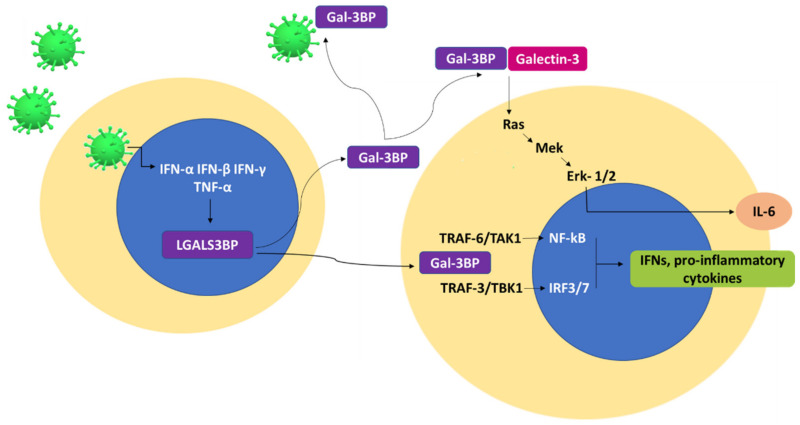
The figure shows some of the known interaction partners and signaling pathways of Gal-3BP, which lead to the expression of proinflammatory cytokines during virus infection. The viral DNA/RNA induces IFNs and TNF-α, that in turn induce Gal-3BP. The protein can act both intra- and extracellularly. Extracellularly, Gal-3BP can interact with galectin-3-inducing RAS-signaling pathway and IL-6 expression. Gal-3BP can also directly interact with virus particles reducing their infectivity. Intracellularly, Gal-3BP acts as a scaffold protein interacting with both TRAF-6/transforming growth factor β-activated kinase 1 (TAK1) and TRAF3/TANK binding kinase 1 (TBK1) complexes which leads to the production of IFNs and proinflammatory cytokines.

Functionally, Gal-3BP intersects various signaling cascade pathways [14], and intra- and extracellular mechanisms have been proposed in which the interaction of the protein with its cellular ligands modulates the expression of proinflammatory and anti-inflammatory cytokines [42,56,57,58] (Figure 3).

Data have been presented to show that secreted Gal-3BP possesses both immunostimulatory and immunosuppressive activities by inducing the release of IL-2 and IL-6 [56,58,59] and suppressing IL-4, IL-5, and IL-13 [60]. Other studies have shown that treatment of CD14+ cells with exogenous Gal-3BP induces the production of IL-1, IL-6, granulocyte-macrophage colony-stimulating factor (GM-CSF), and TNF-α [61] and the induction of proinflammatory genes in human-monocytes-derived macrophages [62]. Even if not all the Gal-3BP interaction partners have been identified, galectin-3 and galectin-1 are the most investigated endogenous ligands [3,63]. The interaction of Gal-3BP with galectin-3 has been demonstrated to be responsible for inducing IL-6 expression and secretion in various cell types, including bone marrow stromal cells (BMSC), neuroblastoma cells, and macrophages. These results are confirmed by the fact that the downregulation of Gal-3BP leads to decreased IL-6 expression. Additionally, data have been presented to show that Gal-3BP/galectin-3-mediated induction of IL-6 comes with a mechanism that involves the Ras-Mek-Erk1/2-signaling pathway [56,57,58]. In addition, secreted Gal-3BP can interact with the dendritic cell (DC)-specific intercellular adhesion molecule (DC-SIGN) with immunosuppressive effects [20,64]. Intracellularly, Gal-3BP has been found to mediate the inactivation and degradation of the transforming growth factor β-activated kinase 1 (TAK1), thus blocking the TAK1-dependent NF-κB-signaling pathway and suppressing inflammation processes [65]. Additionally, Xu et al. [42] demonstrated that intracellular Gal-3BP regulates the production of IFN and inflammatory cytokines, working as a scaffold protein that forms complexes with TRAF6 and TAK1 and with TRAF3 and TANK binding kinase 1 (TBK1), leading to the translocation of the transcription factors NF-κB and IRF3/7 from the cytoplasm to the nucleus. Moreover, Gal-3BP has been demonstrated to exert an antiviral activity by directly interacting with virus components [48], as in the case of adeno-associated viruses, that induces viral particle aggregation and impairment of transduction [66], or HIV-1 Gag that prevents transportation of HIV-1 Gag to the plasma membrane, thus inhibiting HIV-1 virion production [48]. Finally, a direct interaction of Gal-3BP with virus particles has been proposed for SARS-CoV-2 [67,68,69] (Table 1).

## 3. Gal-3BP and SARS-CoV-2 Infection

Recently, studies have demonstrated that Gal-3BP is one of the proteins abundantly expressed in the circulation of patients infected by SARS-CoV-2 [39,40,71,72]. Gal-3BP plasma levels were measured retrospectively in a cohort of 84 hospitalized COVID-19 patients with nonsevere and severe disease. Compared to healthy controls, Gal-3BP levels were noticeably increased in COVID-19 patients (*p* < 0.0001), being higher in severe than in nonsevere patients (*p* < 0.05) [40]. In another study, a longitudinal analysis of hospitalized COVID-19 patients demonstrated that upregulation of Gal-3BP was greater during the early hospitalization and decreased over time [73]. All these studies stated a correlation between Gal-3BP levels and COVID-19 disease severity. Furthermore, patients with severe disease had higher IL-6 plasma levels compared to those with nonsevere disease (*p* < 0.01) [40]. Increased levels of inflammatory cytokines, especially IL-6, have been associated with the risk of developing severe COVID-19 disease [74,75]. At present, the role of Gal-3BP in SARS-CoV-2 infection remains controversial. The overexpression of Gal-3BP in COVID-19 patients has been demonstrated to impair SARS-CoV-2 spike glycoprotein-induced syncytia formation and spike-pseudo-particle transduction efficiency (while it does not affect SARS-CoV-2 spike-pseudo-particle entry), suggesting an antiviral effect of Gal-3BP on SARS-CoV-2 involving intracellular mechanisms [72]. Other studies suggest that the elevated levels of Gal-3BP in COVID-19 patients may have a role in the progression to a severe disease. In keeping with this last hypothesis, increasing evidence suggests Gal-3BP has a key role in the development of the detrimental outcomes of the diseases, including lung fibrosis [39,41]. 

### 3.1. Role of Gal-3BP in Severe COVID-19 Disease and Pulmonary Fibrosis

**Figure 4:**
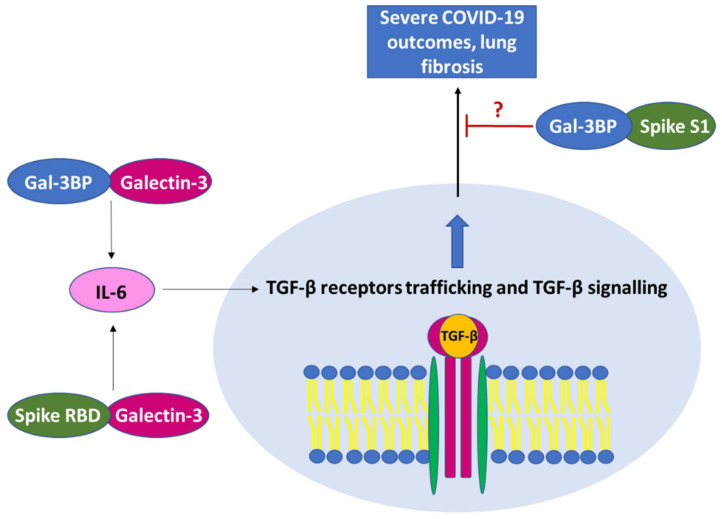
Schematic representation of the possible significance of the interaction between the spike RBD glycans and galectin-3 and between the S1 subunit of spike protein and Gal-3BP in miming and/or blocking the Gal-3BP/galectin-3 signaling. Gal-3BP/galectin-3 interaction leads to increased expression and secretion of IL-6, which has been demonstrated to increase TGF-β signaling and play a role in the development of fibrosis. This profibrotic mechanism resembles that mediated by SARS-CoV-2; indeed, the interaction of Spike RBD glycans with galectin-3 has been demonstrated to upregulate IL-6. This led us to hypothesize that SARS-CoV-2 spike could mimic the Gal-3BP/galectin-3 signaling. Furthermore, in vitro studies demonstrated the possible interaction between Gal-3BP and the S1 galectin-like domain of the spike protein. While there is strong evidence of the antiviral significance of this interaction (see the main text for further details), no studies have been conducted to demonstrate the possibility that it could also act differently. However, the findings of Gal-3BP/S1 spike interaction suggest that it could block the Gal-3BP signaling and the detrimental outcomes of severe COVID-19; thus, we believe that it might be interesting to consider this for further investigations.

The “acute respiratory distress syndrome” (ARDS) is a severe pulmonary complication caused by SARS-CoV-2 virus infection, that in the early pandemic was found in about 5% of COVID patients [76,77]. ARDS has been shown to be mainly the consequence of an exacerbated immune response that leads to a massive release of a specific subset of proinflammatory cytokines, such as TNF-α, IL-2, IL-6, IL-7, IL-10, granulocyte-colony-stimulating factor (G-CSF), interferon-γ-induced protein 10 (IP10), monocyte chemoattractant protein-1 (MCP-1), macrophage inflammatory protein 1 α (MIP-1A) and C-reactive protein (CRP) [78,79,80]. This is accompanied by abnormally increased production and augmented biological activity of anti-inflammatory cytokines, especially TGF-β, as reported in severe COVID-19 disease. TGF-β is a key cytokine in regulating chronic immune reaction in severe COVID-19 and is known to play a crucial role in the development of some peculiar clinical manifestations of the infection, including fatigue, loss of olfactory and taste senses, and in the development of the detrimental pulmonary fibrosis [81,82,83,84,85]. Indirect evidence suggests that Gal-3BP/galectin-3 interaction has a key role in these pathological processes. Indeed, COVID-19 patients who developed pneumonia, particularly those requiring ICU admission, display increased Gal-3BP levels, which are paralleled by increased galectin-3 levels. In addition, patients with high serum levels of galectin-3 (above 35.3 ng/mL) were found to have an increased risk of severe ARDS, admission to ICU, and mortality [39,41]. Furthermore, a positive correlation between galectin-3 and inflammatory markers, including interleukin-6, has been reported by Kuśnierz-Cabala et al. These authors hypothesized that galectin-3 may be involved in severe COVID-19, especially in the development of pulmonary fibrosis [39]. Interestingly, galectin-3, which is an important modulator of acute and chronic inflammation, is the most studied interaction partner of Gal-3BP. The lectin is found in the cytoplasm, in the nucleus, and as a membrane-associated receptor of both normal and malignant cells [86] and plays important roles in signal transduction [87]. Diverse studies reported that during inflammation, galectin-3 loses its proinflammatory functions and promotes tissue healing, inducing the formation of fibrotic tissue [88,89]. Indeed, diverse galectin-3 inhibitors have been reported to counteract fibrotic disorders, including idiopathic pulmonary fibrosis [90]. Further studies reported that galectin-3 induces pulmonary fibrosis by promoting the activity of the transforming growth factor β (TGF-β) [88,89]. Even if the mechanism of action remains still poorly understood, the interaction between Gal-3BP and galectin-3 has been found to be involved in inducing increased expression levels of IL-6 [56]. Interestingly, IL-6 is known to increase trafficking of TGF-β1 receptors to nonlipid raft-associated pools that results in augmented TGF-β signaling [43,44]. These data suggest that the Gal-3BP/galectin-3 axis could be involved in the TGF-β-mediated fibrosis. Interestingly, the putative Gal-3BP/galectin-3 profibrotic mechanism of action resembles those mediated by SARS-CoV-2 in the induction of the detrimental outcome of pulmonary fibrosis, thought to be related to TGF-β increased levels [83,91]. In this regard, a recent study demonstrated that cytokines linked to COVID-19 disease are induced by the S1 subunit of the SARS-CoV-2 spike protein in monocytes. In particular, the S1 subunit of the spike protein, but none of the other spike subunits, induces IL-6, IL-1β, and TNF-α secretion in cultured monocytes [92]. Interestingly, NMR studies showed that the two N-glycans of the receptor-binding domain (RBD) of the spike protein bind to galectin-3 [93], eliciting the possibility that the S1 subunit of spike could interact with galectin-3 with the participation of Gal-3BP. In addition, data showed that the N-terminal domain of SARS-CoV-2 spike protein S1 (i.e., the galectin-like NTD) is highly homologous to galectin-3 [94]. The presence of the galectin-like domain within the NTD of the S1 subunit of the spike protein supports the possibility of Gal-3BP/spike interaction [94] that could possess an antiviral activity. The putative significance of the spike protein’s interaction with Gal-3BP or galectin-3 in miming and/or blocking the effects of the Gal-3BP/galectin-3 signaling is reported schematically in Figure 4.

### 3.2. Interaction of Gal-3BP with SARS-CoV-2 Components and Spike Protein

Studies carried out on various viruses, including HIV-1 and adeno-associated viruses, have demonstrated that one of the mechanisms by which Gal-3BP exerts its antiviral effects is through a direct interaction with viral particles [48]. To date, there are no studies that confirm this mechanism of action also for SARS-CoV-2. However, data from pull-down experiments, conducted on the plasma of COVID-19 ICU patients, demonstrated that Gal-3BP is consistently retrieved together with the SARS-CoV-2 spike protein, strongly indicating that Gal-3BP is a possible binding partner of the viral protein [72]. This has previously been supported by the presence of a galectin-like domain within the NTD of the S1 subunit of the spike protein. Beside the interaction with the spike protein, a Gal-3BP direct interaction with other virus components could be also plausible, since SARS-CoV-2 is an enveloped virus displaying the glycans produced in the infected cells. Indeed, the N-terminal domain of the spike protein, that is homologous to a region of galectin-3, has been demonstrated to bind other glycans including galectin-7 and 8, Siglec-10, macrophage galactose lectin (MGL), and DC-SIGN [93,95], suggesting that Gal-3BP could interact with SARS-CoV-2 also through these molecules. Even if the significance of the putative interaction between Gal-3BP and SARS-CoV-2 components has not yet been solved, data showed that Gal-3BP overexpression is related to the reduction in spike-mediated syncytia formation, and to a decreased spike-pseudo-particle entry. This can be an important issue, since spike-mediated syncytia formation and entry are the most targeted functions of spike protein in the drug discovery field [70]. In addition, a mechanism of SARS-CoV-2 entry has been proposed that involves the galectin-like spike NTD; data supported a dual attachment model for SARS-CoV2, where the spike CTD domain is involved in ACE-2 receptor recognition, while the NTD region binds gangliosides on the cell surface to stabilize viral adhesion [67]. Indeed, studies reported a great affinity between spike NTD and GM1 ganglioside [68,69]; moreover, a high binding affinity has been demonstrated also between GM1 ganglioside and galectin-3 [96], suggesting that galectin-3 inhibitors could impair virus entry [67]. Altogether, these results allow us to speculate on the possibility that Gal-3BP interaction with the galectin-like NTD region of the spike protein could play inhibitory roles in virus entry. Further studies are necessary to confirm Gal-3BP interaction with SARS-CoV-2 components and its significance in SARS-CoV-2 infection.

### 3.3. Clinical Significance of Gal-3BP in SARS-CoV-2 Infection

Despite the advancements made from the beginning of the COVID-19 pandemic to control virus spread and reduce mortality, there is still no effective approach for the prevention or treatment of the severe form of the disease, that often leads to life-threatening complications, such as ARDS. The detection of severity-associated biomarkers is one of the most important tools for the early individuation of patients at risk of developing ARDS, that is crucial for timely treatments [97]. Altered values of various laboratory markers, including increased levels of C-reactive protein, lactate dehydrogenase, serum amyloid A, fibrinogen, D-dimer, adenosine deaminase, and lower levels of lymphocyte, eosinophil, platelet counts, calcium, albumin, albumin/globulin, have been associated with COVID-19 severity [98]. In addition, higher levels of diverse inflammatory cytokines are associated with the disease’s severity, especially IL-6, that has been demonstrated to be one of the most accurate predictors of disease course and mortality [99]. Interestingly, also galectin-3 has been proposed as a potential COVID-19-severity-associated marker. Indeed, studies showed that galectin-3, besides being an independent marker of severity, significantly improves the likelihood to assess the risk of developing a severe outcome when used in combination with other markers such as C-reactive protein and albumin [100], hence the importance of extending the panel of already-known severity-associated clinical markers. In this context, the higher levels of Gal-3BP observed in plasma samples of patients with severe compared to those with nonsevere COVID-19 disease, in addition to the established role of the protein in stimulating IL-6 production and secretion through interaction with galectin-3, makes Gal-3BP a new promising prognostic biomarker of COVID-19 severity. 

## 4. Conclusions

Gal-3BP has been demonstrated to be upregulated during several viral infections, including SARS-CoV-2. Various studies demonstrated a direct correlation between Gal-3BP plasma levels and viral infection progression and/or severity of disease, suggesting a key role of Gal-3BP in modulating immune response to viral pathogens other than a role as a prognosticator. However, the complex biology of Gal-3BP, that encompasses different known and unknown interaction partners and signaling cascades, makes it difficult to elucidate its mechanisms of action, although it could explain the various effects ascribed to the protein. Indeed, both an antiviral activity and a role in inducing disease progression and severe clinical manifestations have been reported for various viral diseases, including COVID-19. Besides modulating immune response to viral pathogens by interacting with various extra- and intracellular cell components, including galectin-3, Gal-3BP has been demonstrated to directly interact with virus components, exerting an antiviral activity. The interaction between Gal-3BP and virus components has been reported for different viruses, including SARS-CoV-2. 

Data have been presented to show that Gal-3BP interacts with the SARS-CoV-2 spike protein and other SARS-CoV-2 envelope components derived from the cell surface of the infected cells. In this context, it also needs to be considered that the type and amount of glycans can differ depending on cell types and vary greatly between individuals, and this could lead to a diverse individual response to the virus. These facts render Gal-3BP interesting in several respects. While the correlation between Gal-3BP levels and disease severity renders the protein a promising prognostic biomarker, its role in inducing detrimental disease outcomes, such as lung fibrosis in severe COVID-19, makes the protein a good candidate as a target to prevent or block pulmonary fibrosis and the other clinical manifestations observed in severe COVID-19. Furthermore, the possible role of Gal-3BP in intersecting SARS-CoV-2 cell targets could be exploited in the attempt to find possible antiviral inhibitors. In addition, a deepened comprehension of Gal-3BP mechanisms of action can be of uttermost importance for an augmented understanding of the molecular mechanisms underlying the host immune response to viral pathogens. In this context, the role of Gal-3BP in COVID-19 could be a useful model to elucidate the role of Gal-3BP in other viral diseases and, considering its putative role in inducing fibrosis, also in other pathologies, including cancer.

Based on the findings discussed in this work, we believe that future investigations should focus on two main purposes: (i) the evaluation of the clinical significance of Gal-3BP as a new severity-associated prognostic marker; and (ii) the search of inhibitors of Gal-3BP/galectin-3 interaction. Furthermore, studies on the correlation between Gal-3BP level in patients with COVID-19 disease and patients with COVID-19 and HIV or other virus infections should be initiated to enhance this field of research.

## Figures and Tables

**Table 1:** Gal-3BP’s interaction partners, mechanisms of action, and reported effects during viral infections.

**Interaction Partner**	**Mechanism of Action**	**Effect**	**References**
Galectin-3	Induction of Ras-Mek-Erk1/2-signaling pathway.	IL-6 expression and secretion.	[56,57,58]
TRAF6/TAK1	Translocation of NF-κB to the nucleus.	Expression of IFN and inflammatory cytokines.	[42]
TRAF3/TBK1	Translocation of IRF3/7 to the nucleus.	Expression of IFN and inflammatory cytokines.	[42]
Viruses	Direct binding with adeno-associated Viruses.	Induces viral particle aggregation and impairment of transduction.	[66]
	Direct binding with HIV-1.	Prevents transportation of HIV-1 Gag to the plasma membrane, inhibiting HIV-1 virion production; reduces the amount of gp120 and gp41 at the plasma membrane.	[14,48]
	Direct binding with SARS-CoV-2 components (i.e., spike protein).	Reduction in spike-mediated syncytia formation, and to a decreased spike-pseudo-particle entry.	[67,68,69,70]
DC-SIGN	DC maturation and fibrocyte differentiation.	Immunosuppression.	[14,20,64]
Unknown	Unknown	Induces Ca^2+^ mobilization; increases the amount of ICAM-I and MHC-I on the cell surface.	[14]
Unknown	Induction of IL-1; IL-2; GM-CSF; and TNF-α.	Immunostimulatory effects.	[56,58,59,61]
Unknown	Suppression of IL-4, IL-5, and IL-13.	Immunosuppressive effects.	[60]

## Data Availability

Not applicable.
